# Targeted next-generation sequencing identifies a homozygous nonsense mutation in *ABHD12*, the gene underlying PHARC, in a family clinically diagnosed with Usher syndrome type 3

**DOI:** 10.1186/1750-1172-7-59

**Published:** 2012-09-02

**Authors:** Tobias Eisenberger, Rima Slim, Ahmad Mansour, Markus Nauck, Gudrun Nürnberg, Peter Nürnberg, Christian Decker, Claudia Dafinger, Inga Ebermann, Carsten Bergmann, Hanno Jörn Bolz

**Affiliations:** 1 Bioscientia Center for Human Genetics, Konrad Adenauer-Str. 17, Ingelheim 55218, Germany; 2 Department of Human Genetics, McGill University Health Center, Montreal, Canada; 3 Department of Obstetrics and Gynecology, McGill University Health Center, Montreal, Canada; 4 Department of Ophthalmology, American University of Beirut, Beirut, Lebanon; 5 Center for Molecular Medicine Cologne, University of Cologne, Cologne, Germany; 6 Cologne Center for Genomics, University of Cologne, Cologne, Germany; 7 Institute of Human Genetics, University Hospital of Cologne, Cologne, Germany; 8 Department of Human Genetics, RWTH Aachen University, Aachen, Germany; 9 Center for Clinical Research, University Hospital of Freiburg, Freiburg, Germany

**Keywords:** Usher syndrome, Deafness, Retinitis pigmentosa, ABHD12, PHARC

## Abstract

**Background:**

Usher syndrome (USH) is an autosomal recessive genetically heterogeneous disorder with congenital sensorineural hearing impairment and retinitis pigmentosa (RP). We have identified a consanguineous Lebanese family with two affected members displaying progressive hearing loss, RP and cataracts, therefore clinically diagnosed as USH type 3 (USH3). Our study was aimed at the identification of the causative mutation in this USH3-like family.

**Methods:**

Candidate loci were identified using genomewide SNP-array-based homozygosity mapping followed by targeted enrichment and next-generation sequencing.

**Results:**

Using a capture array targeting the three identified homozygosity-by-descent regions on chromosomes 1q43-q44, 20p13-p12.2 and 20p11.23-q12, we identified a homozygous nonsense mutation, p.Arg65X, in *ABHD12* segregating with the phenotype.

**Conclusion:**

Mutations of ABHD12, an enzyme hydrolyzing an endocannabinoid lipid transmitter, cause PHARC (polyneuropathy, hearing loss, ataxia, retinitis pigmentosa, and early-onset cataract). After the identification of the *ABHD12* mutation in this family, one patient underwent neurological examination which revealed ataxia, but no polyneuropathy. ABHD12 is not known to be related to the USH protein interactome. The phenotype of our patient represents a variant of PHARC, an entity that should be taken into account as differential diagnosis for USH3. Our study demonstrates the potential of comprehensive genetic analysis for improving the clinical diagnosis.

## Background

Usher syndrome (USH) is an autosomal recessive disorder manifesting in about 10% of children with congenital sensorineural hearing impairment, characterized by additional retinitis pigmentosa (RP). The clinical subtype USH1 presents with severe to profound hearing loss, vestibular impairment and early RP, whereas USH2 is characterized by moderate to severe hearing impairment and RP in adolescence. USH3 is very rare and variable; it may resemble USH1 or USH2, and vestibular dysfunction may be present. Ten genes (including a digenic contributor and modifier, *PDZD7*) have been implicated in USH [1-3]. Clarin-1 (*CLRN1*) has been the only known USH3 gene to date (locus: *USH3A*) until recently, when *HARS*, encoding histidyl-tRNA synthetase, has been proposed as a novel USH3 gene [4]. Moreover, Dad et al. have mapped a condition with clinical overlap to USH3 (RP, progressive hearing impairment, vestibular dysfunction, and congenital cataract) to chromosome 15q22.2-23 [5].

We have identified a consanguineous Lebanese family with an USH3-like phenotype. The patients exhibited sensorineural hearing loss, RP and cataracts, an ocular complication USH patients are predisposed for. Our study aimed at identifying the causative mutation – and potentially the second USH3 gene – in this family by homozygosity mapping and targeted next-generation sequencing (NGS). After the identification of a homozygous truncating mutation in a known disease gene, *ABHD12*, one patient was re-examined and found to display ataxia, reversing the diagnosis to a neurodegenerative disease, PHARC, that is characterized by polyneuropathy, hearing loss, ataxia, retinitis pigmentosa, and early-onset cataract.

## Methods

### Patients

The study was approved by the institutional review board (IRB) at the American University of Beirut and the institutional review board of the Ethics Committee of the University Hospital of Cologne. It was performed in adherence to the tenets of the declaration of Helsinki. Written consent was obtained from all participants. Venous blood samples were obtained for DNA extraction and genomic DNA was isolated following standard protocols.

**Figure 1:**
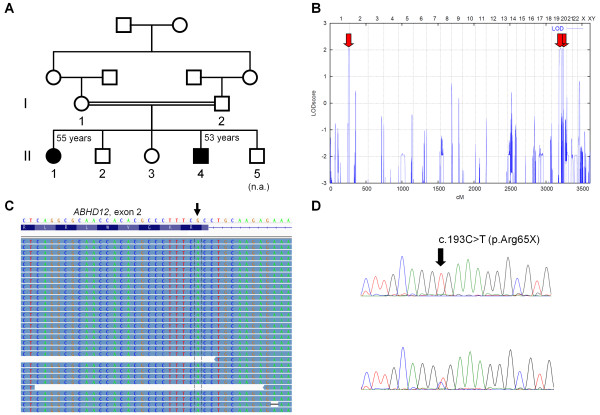
** PHARC family described herein and conducted genetic investigations. A** Pedigree of the family described herein. **B** Graphical view of the LOD score calculation of genomewide SNP mapping. Regions showing homozygosity by descent were identified on chromosomes 1 and 20 (two regions) and are indicated by arrows. **C** Schematic representation of the mapped sequencing reads (reverse strand) visualized with the *Integrative Genomics Viewer* (IGV) for patient II:1. The c.193C > T mutation in exon 2 of *ABHD12* was present in all 87 reads covering this region of the gene (arrow). **D** Sanger sequencing confirmed the homozygous mutation in both patients (upper panel). It was found in heterozygous state in both parents (I:1 and I:2) and in II:2 (lower panel). II:3 was not a carrier.

In both patients (II:1, female, 55 years) and II:4 (male, 53 years; Figure 1A), the diagnosis was established by medical history and detailed evaluation of vision and hearing. Ophthalmological examination consisted of funduscopy, standard ERG, perimetry, measurement of dark adaptation, Farnsworth D-15 color test and determination of visual acuity. II:1 underwent neurological examination after the identification of the homozygous *ABHD12* mutation.

Auditory function of both patients was assessed by pure tone audiometry, speech-audiometry, and tympanometry. Hearing impairment was classified as mild (20–40 dB), moderate (41–70 dB), severe (71–95 dB), or profound (95 dB).

### Linkage analysis

Based on the parental consanguinity and the assumption of autosomal recessive inheritance, we performed genomewide homozygosity mapping using the Affymetrix GeneChip Human Mapping 10 k Array, version 2.0 (Affymetrix, Santa Clara, CA, USA). GRR [6] and PedCheck [7] were used to verify relationships and to identify Mendelian errors. Non-parametric linkage analysis was carried out with MERLIN [8]. Parametric linkage and haplotype analysis were performed using ALLEGRO [9] assuming autosomal recessive inheritance, full penetrance and a disease gene frequency of 0.0001. All data handling was performed using the graphical user interface ALOHOMORA [10]. Graphic output of haplotypes was generated with HaploPainter [11].

### Sequence capture and next-generation sequencing

The coding exons of 378 genes located in the three homozygosity-by-descent (HBD) regions (RefSeq database) corresponded to 2855 regions and 505 kb of sequence, respectively, and were targeted with a customized 385 k sequence capture array (Roche NimbleGen). Genomic DNA from patient II:1 was fragmented (Covaris S2 AFA system) and ligated to sequencing-specific adaptors. Sample was enriched by array hybridization, followed by elution and post-capture amplification by ligation-mediated (LM-) PCR. This amplified DNA was subjected to emulsion PCR (emPCR) and subsequent massively parallel sequencing on a Roche 454 GS FLX platform. A total of 1,182,726 reads were generated corresponding to nearly 400 Mb of sequence information. Approximately 98% of the reads mapped to regions of the hg19 genome, resulting in a 56-fold average depth of coverage for all targeted regions. Less than 1% of the target regions were not covered by at least one unique read, whereas 92% of the regions were covered more than 15-fold. Sequence data for the *ABHD12* gene were compared to the reference sequences NM_001042472.2 and NM_015600.4. The identified *ABHD12* variant was validated by Sanger sequencing.

## Results

### Clinical evaluation

By history, both affected siblings achieved normal psychomotor development including acquisition of speech and language. In both, hearing loss was first noted at the age of 14 years (together with bilateral tinnitus after a severe influenza infection in II:1), but probably occurred earlier. The earliest audiology reports that were available to us referred to investigations performed at the age of 17 years (patient II:1) and 24 years (patient II:4), respectively, both showing moderate-to-severe bilateral neurosensory hearing impairment. Both patients had normal ear canals and tympanic membranes and experienced progressive hearing loss, leading to severe (right) to profound (left) hearing loss. Patient II:1 complained about mild imbalance. A high-resolution CT of the temporal bones and the brain in II:1 was normal. Both patients used hearing aids in the past and received cochlear implants around the age of 35 years.

Nyctalopia became apparent at the age of 18 years in both patients. II:1 underwent sequential bilateral cataract surgery at the age of 26 and 29 years, respectively. Best visual acuity was 20/25 (6/9) bilaterally at the age of 30 years. Dark adaptation, color vision, and ERG were moderately abnormal compared to her brother. Ten years later, at the age of 40 years, funduscopy revealed peripheral retinal changes with fine macular pigmentary changes and best corrected visual acuity of 20/200 (6/60). Last exam at the age of 55 years revealed best corrected visual acuity of 20/400 (6/120) for the right eye and counting fingers for the left eye, left esotropia, 20° of visual fields bilaterally, a cup-disc ratio of 0.9 bilaterally from glaucoma on topical therapy, and fine retinal spicules at the equator.

For II:4, examination records were available from the age of 23 years onward and gave similar results in both eyes. Best corrected visual acuity was 20/100 (6/30) with early posterior subcapsular cataract, vitreous degeneration, marked pallor of optic disc, marked narrowing of retinal vessels, and peripheral retinal pigmentation. Abnormal dark adaptation, constricted visual fields, markedly reduced ERG, and normal Farnsworth D-15 color test supported the clinical diagnosis of USH. Eye exam at the age of 38 years revealed best corrected visual acuity of finger counting at 2 m, moderate posterior subcapsular cataract, severe disc pallor and marked peripheral intraretinal bone spicules in both eyes.

At the most recent examination at the age of 50 years, vision was reduced to light perception on both eyes with moderate posterior capsular cataract, and medically controlled glaucoma. The macular region was atrophic with severe optic atrophy.

II:1 underwent neurological evaluation at the age of 53 years because of a four year history of gait imbalance and writing difficulties for three years. There was a history of tremor since the age of 19 years. Her examination revealed an ataxic gait with poor tandem walking. She had an action tremor with writing cramp and involuntary athetotic movements of her fingers. Her finger to nose exam, muscle tone, and deep tendon reflexes were normal with negative Babinski sign. Brain morphology was normal in a CT of the brain.

In both patients, examination of cranial nerves III, IV, and VI revealed normal eye movements without nystagmus (neither spontaneous nor gaze-induced). Slow pursuit was smooth and saccades were accurate. Trigeminal sensation, corneal reflexes and facial function were intact. In general, disease progression was similar in both cases.

### Linkage analysis and mutation analysis of positional candidate genes

We obtained a maximum parametric LOD score of 2.05 for the three chromosomal regions that showed homozygosity-by-descent (HBD) in II:1 and II:4, on chromosomes 1q43-q44 (8.54 Mb), 20p13-p12.2 (10.48 Mb), and 20p11.23-q12 (19.35 Mb) (Figure 1B). Direct sequencing of all coding exons excluded mutations in the *NINL* gene on chromosome 20p11.21, encoding ninein-like protein which has been shown to interact with the USH protein complex through usherin (USH2A) [12]. Although the causative mutation most likely resides in the longest HBD segment [13], we decided to analyze the coding exons contained in all candidate loci simultaneously.

### Next-generation sequencing

276 high-confidence sequence variations were annotated in the target regions (121 non-synonymous single nucleotide variants (SNVs), 108 synonymous SNVs, 43 frameshift insertions/deletions and 4 in-frame-insertions/deletions). Heterozygous variants, variants annotated as single-nucleotide polymorphism in dbSNP135 and all synonymous variants were neglected. Questionable changes in homopolymer stretches were assessed through visualization with the *Integrative Genomics Viewer*. Finally, three homozygous variants, including one nonsense mutation, remained (Table 1). This nonsense mutation (c.193C > T/p.Arg65X) is located in exon 2 of the *ABHD12* gene (Figure 1C). It was confirmed by Sanger sequencing (Figure 1D) and showed perfect cosegregation with the disease in the family. It is neither present in the databases of the 1000genomes project ( http://www.1000genomes.org), which lists *ABHD12* variants found in 1092 individuals from four different populations, nor in the Exome Variant Server database ( http://evs.gs.washington.edu/EVS).

**Table 1:** Homozygous SNVs without SNP annotation identified by next-generation sequencing of mapped HBD regions

**Gene**	**Refseq**	**cds (ref./var.)**	**Ratio reference/variant**	**AA change**	**rs number**
*ABHD12*	NM_001042472.2 NM_015600.4	c.193C > T	0/85	p.Arg65X	NA
*ID1*	NM_002165	c.34 G > A	1/54	p.Ala12Thr	NA
*RGS7*	NM_002924	c.200 T > C	1/21	p.Ile67Thr	NA

NA, not annotated.

## DISCUSSION

Recent studies indicate a population-prevalence for USH of 1/6000 [14]. Children with USH initially have non-syndromic hearing loss (NSHL): RP manifests in the first (USH1) or second (USH2, USH3) decade of life. At least 10% of the hearing-impaired children carry mutations in USH genes, making USH an important differential diagnosis. Molecular genetic testing can confirm or exclude USH at an early time point, even before the onset of visual problems, and may help limiting detailed ophthalmological follow-up in deaf children to those with USH-causing mutations. Moreover, because certain mutations in USH genes cause hearing impairment without retinal degeneration, USH-causing mutations will be unevitably identified in children with apparently NSHL by massively parallel NGS of all known deafness genes – an approach that will become a diagnostic routine within the next few years.

Mutations in the known USH genes account for 72 – 86% of cases [15,16]. The remainder may be due to mutations far outside the coding regions [17] or large structural rearrangements [18] in these genes that escape detection by genomic DNA amplification and sequencing of the coding exons, and to further genetic heterogeneity. Moreover, USH may be mimicked by clinically overlapping conditions, such as Alström syndrome, or by the co-occurrence of non-syndromic deafness and RP in the same individual [19]. Therefore, the awareness of potential differential diagnoses is important when seeking molecular verification of the clinical diagnosis.

Both patients from our family have deafness, RP and cataracts, all symptoms compatible with USH. Linkage analysis excluded all loci for known USH genes, and targeted NGS revealed an *ABHD12* nonsense mutation segregating with disease in the family. Truncating *ABHD12* mutations have been shown to cause PHARC, a neurodegenerative disease with polyneuropathy, hearing loss, ataxia, RP, and early cataract [20]. II:1 therefore underwent neurological examination which revealed ataxia – a symptom that may be present in USH1 and USH3 due to the affection of vestibular hair cells. Most patients with PHARC and confirmed *ABHD12* mutations had ataxia, and these patients had cerebellar atrophy or peripheral polyneuropathy or both [20]. There were no obvious signs of polyneuropathy in patient II:1, and no indication of cerebellar atrophy in the cranial CT scan. However, her balance problems could result from polyneuropathy that remained undetected because detailed investigation of the peripheral nerves had been omitted as long as USH was the clinical diagnosis. II:4 did not complain about balance problems, indicating that ataxia was either very mild or not present. Unfortunately, this patient was not available anymore for detailed clinical follow-up. However, given the homozygous *ABHD12* nonsense mutation segregating with the disease, the phenotype in our family can be considered a variant of PHARC.

In retinal photoreceptor cells, the USH protein interactome presumably plays a role in transport, trafficking and synaptic function; in the inner ear, the USH protein interactions are important for hair cell development, maintenance, and for tip link formation and hence mechanotransduction [21-23]. The only known USH3 protein known, clarin-1, has recently been shown to be part of this interactome as well [24]. The PHARC protein ABHD12 hydrolyzes 2-arachidonoyl glycerol, an endocannabinoid lipid transmitter that acts on cannabinoid receptors CB1 and CB2. The pathway affected in PHARC is not yet known to be related to the USH protein complex. As can be expected because of the RP component in PHARC, *ABHD12* is expressed in the retina [20], but no details are available on inner ear expression. Further studies are needed to determine the expression of ABHD12 on the cellular and subcellular level in both sensory systems. The investigation of potential interactions of ABHD12 with the known USH proteins will be crucial to find out if the clinical overlap of PHARC and USH is based on a functional relationship between these proteins.

## Conclusions

We suggest that PHARC should be taken into account as differential diagnosis for USH, especially in “USH3-like” patients with progressive hearing loss and balance problems. Comprehensive genetic analysis, mainly by NGS-based approaches, will increasingly be helpful in correcting the diagnosis (reverse phenotyping) [25] and thereby improve patient management.

## Competing interests

TE, MN, CD, CB and HJB are employees of Bioscientia, which is part of a publicly traded diagnostic company.

## Authors’ contributions

RS and AM recruited the family described herein and collected the clinical data. GN and PN performed the linkage analyses. TE, ChD, CD and IE performed the sequencing analyses and statistical interpretation. RS, MN, CB and HJB oversaw all aspects of the research. HJB initiated, planned and coordinated the study. HJB, RS and TE wrote the manuscript. All authors read, edited and approved the final version of the manuscript.
